# Cancer profile among medical evacuees from São Tomé and Príncipe: a descriptive study (2019–2025)

**DOI:** 10.3332/ecancer.2026.2093

**Published:** 2026-03-12

**Authors:** Liudmila Castelo David, Teresa Mota Garcia, Isaulina Barreto, Esperança Carvalho, Laurinda Barreto, Clara Aleydis, Laurinda Coelho, Lúcio Lara Santos

**Affiliations:** 1 Hospital Ayres de Menezes, São Tomé e Príncipe; 2 Grupo de Epidemiologia, Resultados, Economia e Gestão em Oncologia, Centro de Investigação do Instituto Português de Oncologia do Porto (CI-IPOP)/RISE@CI-IPOP (Health Research Network)/Porto Comprehensive Cancer Center (Porto.CCC), 4200-072 Porto, Portugal; 3 Serviço de Epidemiologia, Instituto Português de Oncologia do Porto (IPO-Porto), 4200-072 Porto, Portugal; 4 Grupo de Patologia e Terapêutica Experimental do Centro de Investigação do IPO Porto (CI-IPOP), Instituto Português de Oncologia, 4200-072 Porto, Portugal; 5 Escola de Medicina e Ciências Biomédicas, Universidade Fernando Pessoa, 4200-072 Porto, Portugal

**Keywords:** neoplasms/epidemiology, medical evacuation, São Tomé and Príncipe, medical oncology, delivery of health care, developing countries

## Abstract

**Background:**

São Tomé and Príncipe, a small island developing state, relies heavily on medical evacuations for specialised cancer care. This country does not yet have a population-based cancer registry. Therefore, limited data exist on cancer epidemiology in this population.

**Objective:**

To characterise the profile of cancer among patients evacuated from São Tomé and Príncipe for medical treatment abroad.

**Methods:**

Descriptive study of medical evacuation records from the Ministry of Health’s Patient Evacuation Board (2019–2025). Descriptive statistics were reported for patient demographics, year of evacuation, primary diagnosis, referring speciality, number of evacuations per patient and if travelled with an escort.

**Results:**

Of 1,066 total evacuations, 413 (38.7%) were cancer-related. Cancer patients had a mean age of 48.3 ± 18.7 years with female predominance (58.6%). Breast cancer was most frequent (24.7%), followed by prostate (17.9%) and cervical cancer (9.4%). Most patients (76.9%) required single evacuation, with cancer patients more likely to have multiple evacuations.

**Conclusion:**

Cancer represents a substantial burden among medical evacuees, with patterns suggesting opportunities for enhanced prevention and early detection programs. The high evacuation rate highlights critical gaps in local oncological capacity. Establishing a population-based cancer registry would enhance epidemiological data and inform public health strategies.

## Background

Cancer incidence is rising globally, with low- and middle-income countries experiencing disproportionate challenges in providing adequate oncological care [1, 2]. Small Island Developing States (SIDS) face unique healthcare challenges due to geographic isolation, limited resources and small population sizes that make specialised medical services economically unfeasible [3].

**Figure 1.**
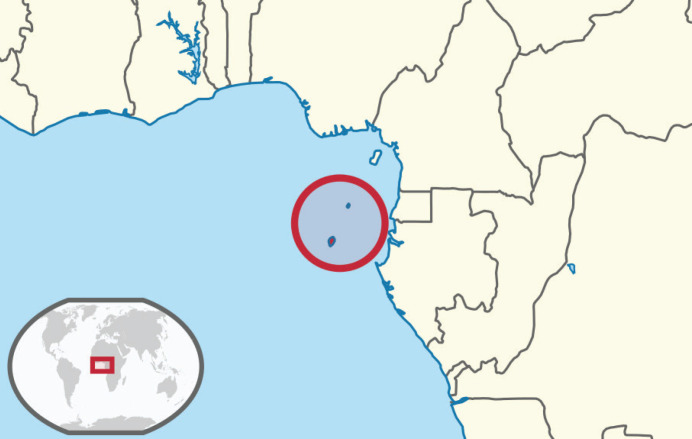
Map of São Tomé. Source: Wikimedia commons.

São Tomé and Príncipe, a SIDS located in the Gulf of Guinea (Figure 1) with approximately 220,000 inhabitants, exemplifies these challenges.

According to GLOBOCAN estimations, São Tomé and Príncipe was estimated to have an age-standardised incidence rate of 104.9 per 100,000 inhabitants in 2022 – a similar rate to neighbouring countries [4]. Also, estimations identify prostate, breast, cervical, lung and stomach cancers to be the most frequent in the country [4].

However, the country currently lacks oncology specialists. In 2023, there were 495 nurses and 154 physicians, making an average of 2.2 nurses and 0.69 medical doctors per 1,000 people [5] – lower than the world average of 3.9 and 1.8, respectively [6, 7]. Among the local physicians, 31 were specialists of Anaesthesiology, Internal Medicine, Paediatrics, General Surgery, Gynaecology and Obstetrics Orthopaedics, Otorhinolaryngology, Ophthalmology, Urology and Psychiatry [5]. Because of this, the country’s healthcare system relies on a medical evacuation program to provide specialised oncology care unavailable locally.

Medical evacuations refer to the transfer of patients from São Tomé and Príncipe to Portugal, by bilateral agreements in the health sector between the Portuguese Speaking African Countries and Portugal, signed after the countries’ independence.

Because these medical evacuations require a registry, this creates a natural surveillance system for complex medical conditions requiring advanced treatment. This is particularly interesting considering that cancer burden data from São Tomé and Príncipe is limited because there is no established population-based cancer registry. Also, the data available from the International Agency for Research on Cancer provides only estimations of cancer burden, based on rates from neighbouring countries or registries in the same area [4]. Therefore, medical evacuation records provide a unique opportunity to examine cancer patterns among patients.

Furthermore, this is the first study to our knowledge that takes advantage of medical evacuation records to provide data to profile cancer, since the only studies of this topic are qualitative studies about the dysfunctions of this process [8–10]

This study aims to characterise the profile of cancer among patients evacuated from São Tomé and Príncipe for medical treatment abroad, providing insights into the cancer burden and challenges faced by the healthcare system in this understudied population.

## Methods

This is a descriptive study to report medical evacuation records from the Ministry of Health’s Patient Evacuation Board in São Tomé and Príncipe. All records from January 2019 to May 2025 were included.

Data was extracted from official evacuation records maintained by the Ministry of Health. Variables included patient demographics (age and sex), year of evacuation, primary diagnosis, referring speciality, number of evacuations per patient, and if travelled with an escort. Cancer cases were defined as patients with primary clinical diagnoses of malignant neoplasms.

Descriptive statistics were calculated using absolute and relative frequencies for categorical variables, and measures of central tendency and dispersion for continuous variables.

This study was reported in accordance with the Strengthening the Reporting of Observational Studies in Epidemiology (STROBE) guidelines for observational studies [11].

This study was approved by the country’s Health Ministry, considering the absence of an Ethics Committee and giving that all efforts were made to ensure anonymity. Only aggregated data is used and no identifying information is included (namely, unique case primary diagnosis).

## Results

During the study period, 1,066 patients were evacuated for medical treatment. Of these, 413 (38.7%) were patients with cancer. Table 1 presents the demographic and evacuation characteristics for overall and cancer cases. The mean age of all evacuated patients was 40.8 years (SD = 22.7). Cancer patients presented with a higher mean age of 48.3 years (SD = 18.7). Females represented the majority in both groups, being 56.5% of the total evacuation group and 58.6% among cancer patients.

Evacuations showed a gradual increase from 2020 to 2024 (from 13.2% to 20.4% of all patients), despite the highest proportion of cancer-related evacuations being in 2022 and 2023 (21.1% and 21.5%, respectively). Cancer patients accounted for the majority of evacuations in 2021 (88.6% of all evacuations) and 2022 (62.1%).

A higher proportion of cancer patients (48.9%) travelled alone compared to the overall group (42.5%). Most patients underwent a single evacuation in both groups (76.9% in overall patients; 72.4% in cancer patients). However, cancer patients were more likely to have multiple evacuations, with 16.9% undergoing three or more, compared to 11.6% in the total group.

Referrals came from a wide range of specialties, with general surgery (12.4%), orthopaedics (10.9%) and cardiology (8.1%) being the most common referring specialties in the overall group. Among cancer patients, general surgery (12.1%), urology (12.3%) and gynaecology and obstetrics (9.2%) were the most frequent. Radiology accounted for 7.5% of cancer referrals, compared to 2.9% in the total group.

Among the 413 cancer cases evacuated (Table 2), women accounted for the majority of cases (58.6%) and had a lower mean age (46.8 ± 17.8 years), when compared to males (50.3 ± 19.8 years). Breast cancer (24.7%) was the most frequent diagnosis, followed by prostate (17.9%) and cervical cancers (9.4%).

Among female cancer patients, breast cancer represented 40.5% of cases, while cervical cancer represented 16.1% of cases. Among male cancer patients, prostate cancer represented 42.9% of cases.

Other diagnoses included brain tumours (6.3%), sarcomas (5.6%) and skin cancers (2.9%), with brain and skin cancers being more incident among males (7.1% and 5.3%, respectively) than females (8% and 1.2%, respectively).

Table 3 shows the most frequent primary diagnoses in all evacuated patients and the medical specialties involved in patient care.

Among the 1,066 patients who underwent medical evacuation, the most frequent primary diagnoses were cancer-related. Breast cancer was the most frequent diagnosis, accounting for 9.6% of cases, followed by prostate cancer (6.9%). Frequent non-oncological conditions were arthrosis/joint surgery (4.5%), renal failure (3.8%) and disc disease (2.3%).

Other cancer diagnoses included cervical cancer (3.7%), brain cancer (2.4%) and sarcoma (2.2%). Traumatic conditions such as fractures and trauma each represented 2.0% of cases, while congestive heart failure and heart valve disease were also present at similar rates (2.0% and 2.2%, respectively).

In terms of medical specialties involved in patient care, oncology was the most frequently engaged, involved in 38.7% of cases. Orthopaedics (15.9%) and cardiology/vascular surgery (15.0%) were also commonly involved.

## Discussion

This study provides a 6-year overview of medical evacuations from São Tomé and Príncipe, with a focus on cancer-related cases.

Among the 1,066 patients evacuated, cancer accounted for a substantial proportion (38.7%), highlighting the significant burden of oncological diseases within the evacuated population. The predominance of cancer-related evacuations in 2021 and 2022 (88.6% and 62.1%, respectively), even amid the COVID-19 pandemic, underscores the critical need for specialised cancer care and the limitations of local healthcare infrastructure in managing oncological conditions.

The difference between GLOBOCAN estimates for São Tomé and Príncipe – which identify prostate, breast, cervical, lung and stomach cancers as the most prevalent – and the findings of this study – which highlight breast, prostate, cervical, brain and sarcoma cancers as the most frequent – underscores the critical need for a National Cancer Registry. The Registry would allow for more accurate and context-specific data, improving the reliability of cancer statistics and informing more effective public health policies and resource allocation.

The mean age of cancer patients (48.3 years) is lower than in high-income countries, suggesting potential genetic predisposition, environmental exposure or limited access to screening programs [1, 2, 4, 12]. The predominance of breast, prostate and cervical cancers, conditions often linked to genetic and lifestyle factors, further supports this hypothesis [13, 14]. This finding prompts further investigation on risk factors and emphasises the need for age-appropriate screening strategies. This is particularly important for cervical cancer, since the observed high proportion (9.3% of all cancer cases) exceeds rates seen in countries with established screening programs [15, 16]. However, it is worth noting that despite the country currently lacking a formal screening program, a national HPV vaccination program has been in place since 2019, targeting girls aged 10 years.

Sex-specific trends revealed that women constituted the majority of cancer cases (58.6%), with breast and cervical cancers being the most prevalent. Among men, prostate cancer was dominant, accounting for 43.5% of male cancer cases. These findings align with global patters, but call for gender-specific prevention and screening strategies [1, 2, 4].

Regionally, the high burden of cervical cancer aligns with sub-Saharan Africa’s epidemiology, where it remains the leading cause of cancer death among women [17, 18].

Cancer patients were more likely to need multiple evacuations (16.9% had three or more), reflecting the complexity of ongoing treatment and the logistical challenges involved. The involvement of specialties such as general surgery, urology and radiology in cancer referrals further illustrates the multidisciplinary nature of cancer care.

Besides highlighting the importance of a Cancer Registry, this study also highlights the need to invest in oncology treatment in São Tomé and Príncipe. The country currently lacks oncology specialists and facilities to provide advanced surgeries, systemic therapies or radiotherapy. As a result, patients must be medically evacuated abroad.

Each medical evacuation has an average medical cost of approximately €3,500 (covered by Portugal [19]), and travel-related expenses for patients and escorts can go to €225,000 yearly (covered by São Tomé and Príncipe). Accommodation expenses are covered by the individuals themselves. This situation places a significant financial burden. Therefore, reducing reliance on external care would alleviate the financial burden and also improve access to timely treatment for patients within the country.

This study has two key limitations: selection bias, due to the exclusion of patients too ill to travel or with advanced disease deemed untreatable abroad; and diagnostic gaps resulting from limited histopathological confirmation caused by the absence of local diagnostic capacity.

Future research should explore the long-term outcomes and assess the cost-effectiveness of medical evacuation as a strategy for managing complex diseases as opposed to investing in local early detection and treatment capacity.

## Conclusion

Firstly, this is the first study that uses medical evacuation records to provide with cancer data in a country where GLOBOCAN can only provide with estimates from neighbouring countries. Secondly, the findings highlight the substantial burden of cancer among medical evacuees from São Tomé and Príncipe. These conclusions enhance the need for a population-based cancer registry to provide with better epidemiological data and inform public health strategies, potentially serving as a model for other SIDS facing similar healthcare challenges.

## Conflicts of interest

The author(s) declare that they have no conflicts of interest.

## Funding

No specific funding was received for this study.

## Author contributions

LCD, IB, EC, LB, LC, CAA: data collection and management

TMG and LLS: study design and writing of the manuscript

LCD, IB, EC, LB, LC, CAA, LLS: revision of the manuscript

## Figures and Tables

**Table 1.** Characteristics of evacuated patients, overall and cancer cases.

	Total evacuations	Cancer cases
	**(*N* = 1,066)**	**(*N* = 413)**
Age		
Mean (SD)	40.8 (22.7)	48.3 (18.7)
Missing	24 (2.3%)	9 (2.2%)
Sex, *n* (%)		
Female	602 (56.5%)	242 (58.6%)
Male	463 (43.4%)	170 (41.2%)
Missing	1 (0.1%)	1 (0.2%)
Year of evacuation, *n* (%)		
2019	213 (20.0%)	51 (12.3%)
2020	141 (13.2%)	39 (9.4%)
2021	70 (6.6%)	62 (15.0%)
2022	140 (13.1%)	87 (21.1%)
2023	198 (18.6%)	89 (21.5%)
2024	218 (20.4%)	56 (13.6%)
2025 (until May)	86 (8.1%)	29 (7.0%)
Travelled with escort, *n* (%)		
Yes	547 (51.3%)	156 (37.8%)
No	453 (42.5%)	202 (48.9%)
Missing	66 (6.2%)	55 (13.3%)
Number of evacuations, *n* (%)		
1 time	820 (76.9%)	299 (72.4%)
2 times	112 (10.5%)	39 (9.4%)
≥ 3 times	124 (11.6%)	70 (16.9%)
Missing	10 (1.0%)	5 (1.2%)
Referring specialty, *n* (%)		
Cardiology	86 (8.1%)	-
General surgery	132 (12.4%)	50 (12.1%)
Maxillofacial surgery	19 (1.8%)	6 (1.5%)
Vascular surgery	1 (0.1%)	-
Stomatology	3 (0.3%)	1 (0.2%)
Gastroenterology	22 (2.0%)	12 (2.9%)
Ginecology and obstetrics	42 (3.9%)	38 (9.2%)
Infectious diseases	3 (0.3%)	1 (0.2%)
Internal medicine	66 (6.2%)	20 (4.8%)
Oftalmology	29 (2.7%)	9 (2.2%)
Orthopedics	116 (10.9%)	12 (2.9%)
Otorhinolaryngology	15 (1.4%)	16 (3.9%)
Pediatrics	5 (0.5%)	2 (0.5%)
Radiology	31 (2.9%)	31 (7.5%)
Urology	58 (5.4%)	51 (12.3%)
Missing	438 (41.1%)	164 (39.7%)

**Table 2.** Characteristics of evacuated cancer patients.

	Cancer cases	Female cases	Male cases
	(N = 413)	(N = 242)	(N = 170)
Age			
Mean (SD)	48.3 (18.7)	46.8 (17.8)	50.3 (19.8)
Missing	9 (2.2%)	7 (2.9%)	2 (1.2%)
Diagnosis			
Breast	102 (24.7%)	98 (40.5%)	4 (2.4%)
Prostate	74 (17.9%)	-	74 (43.5%)
Cervix	39 (9.4%)	39 (16.1%)	-
Brain	26 (6.3%)	14 (5.8%)	12 (7.1%)
Sarcoma	23 (5.6%)	14 (5.8%)	9 (5.3%)
Skin	12 (2.9%)	3 (1.2%)	9 (5.3%)
Stomach	10 (2.4%)	6 (2.5%)	4 (2.4%)
Lymphoma	10 (2.4%)	6 (2.5%)	4 (2.4%)
Bladder	9 (2.2%)	4 (1.7%)	5 (2.9%)
Uterus	8 (1.9%)	8 (3.3%)	-
Thyroid	7 (1.7%)	4 (1.7%)	3 (1.8%)
Lung	7 (1.7%)	4 (1.7%)	3 (1.8%)
Kidney	7 (1.7%)	4 (1.7%)	3 (1.8%)
Other	79 (19.1%)	37 (15.3%)	41 (24.1%)

**Table 3.** Primary diagnosis and medical specialties involved in patient care in overall evacuated patients.

	Total
	(N = 1,066)
Primary diagnosis (Top 12)	
Breast cancer	102 (9.6%)
Prostate cancer	74 (6.9%)
Arthrosis/Joint surgery	48 (4.5%)
Cervix cancer	39 (3.7%)
Renal failure	41 (3.8%)
Brain cancer	26 (2.4%)
Disc disease	24 (2.3%)
Fracture	21 (2.0%)
Congestive heart failure	21 (2.0%)
Sarcoma	23 (2.2%)
Heart valve disease	23 (2.2%)
Trauma	21 (2.0%)
Fracture	21 (2.0%)
Congestive heart failure	21 (2.0%)
Medical specialties involved in patient care	
Oncology	413 (38.7%)
Orthopaedics	169 (15.9%)
Cardiology/Vascular surgery	160 (15.0%)
Nephrology	73 (6.8%)
Neurology	49 (4.6%)
Gastroenterology	36 (3.4%)
Ophthalmology	36 (3.4%)
Haematology	34 (3.2%)
Maxillofacial surgery	14 (1.3%)
Infectious diseases	13 (1.2%)
Pneumology	12 (1.1%)
Gynaecology and obstetrics	12 (1.1%)
Endocrinology	10 (0.9%)
Otorhinolaryngology	10 (0.9%)
Rheumatology	10 (0.9%)
Urology	6 (0.6%)
Dermatology	6 (0.6%)
Neonatology	2 (0.2%)
Allergology	1 (0.1%)
